# Altered long noncoding RNA profile after intracerebral hemorrhage

**DOI:** 10.1002/acn3.50894

**Published:** 2019-09-26

**Authors:** Jeong‐Min Kim, Jangsup Moon, Jung‐Suk Yu, Dong‐Kyu Park, Soon‐Tae Lee, Keun‐Hwa Jung, Kon Chu

**Affiliations:** ^1^ Laboratory for Neurotherapeutics Biomedical Research Institute Seoul National University Hospital Seoul Republic of Korea; ^2^ Department of Neurology Chung‐Ang University Medical Center Seoul Republic of Korea; ^3^ Department of Neurology College of Medicine Seoul National University Hospital Seoul National University Seoul Republic of Korea

## Abstract

**Objective:**

We investigated the expression pattern of long noncoding RNAs (lncRNA) and messenger RNAs (mRNA) from two different intracerebral hemorrhage (ICH) rat models, and performed gene ontology and gene/protein interaction analyses.

**Methods:**

We harvested hemorrhagic brain 1, 3, and 7 days after ICH induction by stereotactic collagenase injection. We performed microarray analyses with Agilent array platform to compare the expression of lncRNA and mRNAs from hemorrhagic and normal brains. The RNA expression patterns were also examined from the autologous blood injection ICH model at days 1 and 3, and significantly altered lncRNAs from two ICH models were validated by quantitative reverse transcriptase‐polymerase chain reaction. Gene ontology analysis and pathway analysis were performed with differentially expressed mRNAs after ICH. Gene and protein interaction analysis was performed to elucidate the functional role of upregulated lncRNA in neuronal damage.

**Results:**

Among the 13,661 lncRNAs studied, 83, 289, and 401 lncRNAs were significantly elevated after 1, 3, and 7 days after collagenase‐induced ICH, respectively. NR_027324, or H19, was the most upregulated lncRNA after 1 day from the two ICH models and its elevation persisted until the 7th day. Gene ontology analysis revealed that immune‐related biological processes such as immune response, immune system process, and defense response were upregulated from both ICH models. Gene and protein interaction study demonstrated that NR_027324 was closely related to the type I interferon signaling pathway.

**Interpretation:**

This study illustrates the dynamic expression pattern of the lncRNA profile following ICH, and that H19 is the most consistently upregulated lncRNA after ICH.

## Introduction

Intracerebral hemorrhage (ICH) is a devastating stroke subtype with high mortality and profound neurological deficit for survivors.^1^ Although the incidence of ICH is decreasing in developed countries due to effective hypertension management, its incidence in the rest of the world is still considerable, and an effective treatment strategy against ICH does not exist yet.^1^, ^2^ Recent randomized controlled trials have failed to show clinical efficacy of rapid blood pressure lowering, a hemostatic agent, or surgical treatment for acute ICH.^2^ The novel treatment strategy based on the mechanism of neuronal damage after ICH is a plausible option which can be successfully translated to clinical practice.

Long noncoding RNA (lncRNA) is a noncoding RNA with more than 200 base pairs without translational activity; nevertheless, recent studies have shown that they can control gene expression by modulating transcription and translational processes.^3^ The role of lncRNA has been studied not only from neoplastic disorders but also from various neurological diseases such as dementia, epilepsy, and cerebral ischemia to understand disease processes and their possible biomarker/therapeutic implications.^4^, ^5^, ^6^ However, no studies have been conducted to disclose the temporal expression pattern of lncRNAs from an acute hemorrhagic stroke model. The analysis of lncRNA expression pattern and its relationship with messenger RNA (mRNA) expression may provide a chance to discover therapeutic targets based on the distinct neuronal injury mechanism after ICH. Therefore, we investigated the expression patterns of lncRNAs from two different ICH models and performed gene ontology/pathway analysis and gene/protein interaction analysis.

## Methods

### Intracerebral hemorrhage model

**Figure 1:**
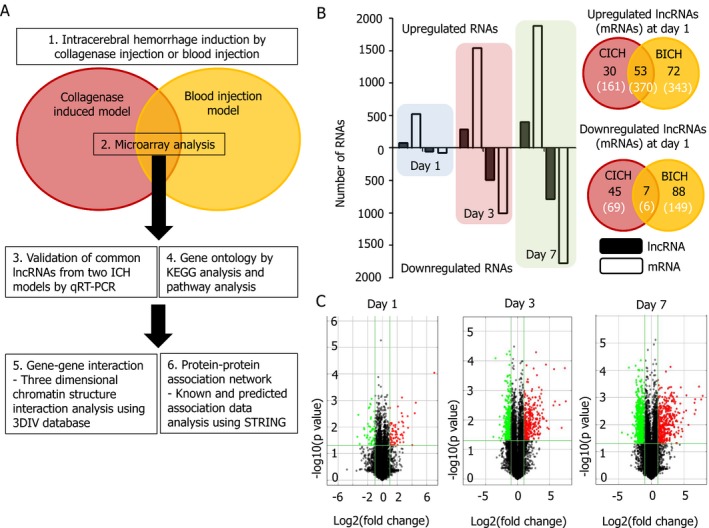
Overall study design and the expression pattern of RNAs in a collagenase‐induced intracerebral hemorrhage model. (A) The flow diagram summarizes the present study scheme, which analyzes the expression patterns of lncRNA and mRNA from two different ICH models followed by gene ontology analysis, pathway analysis, and gene/protein interaction analysis. (B) Temporal trends of RNA expression reveal that both lncRNAs and mRNAs showed robust expression change as time passes from collagenase induced model. Comparison with blood injection model revealed that 53 lncRNAs and 370 mRNAs were concordantly upregulated at day 1, and 7 lncRNAs and 6 mRNAs concordantly downregulated (black number = the number of lncRNAs, white number in bracket = the number of mRNAs). (C) Volcano plots show an increasing trend in significantly altered lncRNAs with more than a twofold change and *P* < 0.05 from collagenase‐induced hemorrhage model.

This study was conducted in accordance with the International Animal Care and Use Committee of Seoul National University Hospital; overall study flow is summarized in Figure 1A. The experimental ICH model was induced using adult male Sprague‐Dawley rats (Daehan Bio, Seoul, Korea) by stereotactic injection of bacterial collagenase type VII after anesthetization with ketamine injection, as described previously (injection point: 3.0 mm left lateral to the midline, 0.2 mm posterior to bregma, 6.0 mm in depth below the skull).^7^ We also constructed another ICH model by injection of 300 *μ*L blood at the same coordinate that collagenase was infused. All surgical procedures were performed under anesthesia to prevent unwanted suffering and the animals were raised with a 12‐h light/dark cycle in a specific pathogen‐free facility with free access to food and water. Control rats did not undergo any surgical procedure. Animals received an intraperitoneal injection of 1% ketamine (30 mg/kg) and xylazine hydrochloride (4 mg/kg) before sacrifice, and striatum was selected for RNA analysis.^8^ The RNA expression levels in the brain after 1, 3, and 7 days from collagenase‐induced ICH (CICH) model and after 1 and 3 days from blood injection ICH (BICH) model were compared to those from a normal healthy brain to evaluate the lncRNA expression change during the acute stage. Each time point consists of four rats; RNA samples from two rats were combined to minimize variability before microarray analyses.

### RNA extraction and analysis

LncRNA microarray analysis was performed by Rat LncRNA Microarray v2.0 (Arraystar, Rockville, Maryland), which contains about 13,611 lncRNAs and 24,626 protein coding transcripts, in accordance with the manufacturer's standard protocols. First, total RNA from each sample was quantified, and its integrity was assessed by standard denaturing agarose gel electrophoresis. Agilent Array platform was employed, and each sample was amplified and transcribed into fluorescent cRNA along the entire length of the transcripts without 3' bias utilizing a random priming method with the Arraystar Flash RNA Labeling Kit (Arraystar, Rockville, Maryland). The labeled cRNAs were hybridized onto the Rat LncRNA Microarray v2.0, and the arrays were scanned after washing the slides. Agilent Feature Extraction software version 11.0.1.1 (Agilent Technologies, Santa Clara, CA) was used to analyze acquired array images, and quantile normalization and subsequent data processing were performed with the GeneSpring GX v12.1 software package (Agilent Technologies, Santa Clara, CA). Differentially expressed lncRNAs and mRNAs with statistical significance between the two groups were identified through volcano plot and fold change filtering, where statistical significance was defined as a fold change ≥ 2.0 and *P*‐value < 0.05. False discovery rate by Benjamini–Hochberg procedure with significance threshold <0.05 was applied to overcome multiple comparison problem. Hierarchical clustering with heatmap was performed to show distinguishable lncRNA expression pattern according to the three different time points from CICH model, which helps to hypothesize the relationships among the samples based on the similarities of their expression levels. The RNA expression levels from the BICH models were also compared with the normal brain by the same methods.

The significantly altered lncRNAs from the two different ICH models were selected and further confirmed by quantitative real‐time polymerase chain reaction (qRT‐PCR). For each sample, the relative amount of the target gene is determined by calculating the ratio between the target lncRNA and GAPDH concentration derived from the standard curve. The sequences of each primer are provided in Table S1. The subclasses of lncRNAs were categorized into six groups according to their relationship with coding sequence: (1) exon sense‐overlapping, the lncRNA's exon is overlapping a coding transcript exon on the same genomic strand; (2) intronic, the lncRNA is overlapping the intron of a coding transcript on the same genomic strand; (3) natural antisense, the lncRNA is transcribed from the antisense strand and overlapping with a coding transcript; (4) nonoverlapping antisense, the lncRNA is transcribed from the antisense strand without sharing overlapping exons; (5) bidirectional, the lncRNAs are oriented head‐to‐head to a coding transcript within 1000 base pairs, (6) intergenic, there are no overlapping or bidirectional coding transcripts nearby the lncRNA, based on recent lncRNA registries (Emsembl, RefSeq, lncRNAdb). The location, size, and possible interaction information with the protein‐coding gene were also gathered from the aforementioned registries.

### Pathway analysis and gene ontology analysis

Gene ontology (GO) and pathway analysis were performed to investigate the potential roles of differentially expressed mRNAs after ICH. The GO categories were derived from Gene Ontology (http://www.geneontology.org) which describes gene attributes in biological processes, cellular components, and molecular function. The pathway analysis was based on the Kyoto Encyclopedia of Genes and Genomes (KEGG, http://www.genome.jp/kegg) database and the enrichment score of different pathways was calculated by –Log (*P*‐value). In both analyses, the *P*‐value, which denoted the significance of each categorization, was calculated and the statistical significance was defined when *P*‐value <0.05.

### Gene and protein interaction analysis

It is hard to clarify how lncRNAs regulate coding genes based solely on their sequence information because noncoding RNAs can influence nearby gene expression by affecting enhancer function or by spatial conformational change.^9^ Co‐expression analysis, such as high‐throughput chromatin conformation capture which can detect long‐range chromatin interactions, enables extensive genome‐wide investigation of both coding and noncoding genes.^10^ 3DIV (http://kobic.kr/3div) is a database which provides a list of long‐range chromatin interactions of any queried locus after normalizing genomic distance and visualizes the listed interactions surrounding the target of interest using a browsing system.^10^ 3DIV database consists of the following three major modules, interaction table, interaction visualization, and comparative interaction visualization, which effectively visualizes distant chromatin interaction patterns surrounding the target gene.^10^ We explored the protein‐coding genes which were predicted to have significant interaction with derived lncRNA from ICH models using the 3DIV database browser.

Among those coding sequences derived from 3DIV database analysis, we selected the genes with elevated mRNA from microarray after ICH and investigated the interaction of those proteins from the STRING database version 10.5 (http://string-db.org) to simulate the functional consequence of elevated lncRNA after ICH. STRING is a database of known and predicted protein–protein interactions to enhance a system‐wide understanding of cellular function derived from the following seven domains: curated database, experimentally determined, gene neighborhood, gene fusions, gene co‐occurrence, text mining, and co‐expression.^11^

## Results

### LncRNAs expression pattern after ICH

Among the 13,661 lncRNAs and 24,626 mRNAs studied via microarray analyses from the CICH model, the number of altered RNAs showed an increasing tendency as time passed (Fig. 1B). The numbers of significantly upregulated lncRNAs were 83, 289, and 401, while the numbers of significantly downregulated lncRNAs were 52, 489, and 786 on the 1st, 3rd, and 7th day, respectively (Fig. 1C). The numbers of significantly elevated lncRNAs from the BICH model on the 1st and 3rd day were 125 and 104, and the numbers of significantly depressed lncRNAs were 95 and 84. The significantly altered lncRNAs with false discovery rate < 0.05 were only detected at 7th day from CICH model: two elevated lncRNAs (XR_593156 and XR_590043) and seven downregulated ones (XR_593346, XR_598840, XR_590087, ENSRNOT00000037029, XR_593035, XR_347800, and XR_590198). The entire lncRNA and mRNA expression levels from all the study groups are illustrated in Tables S2 and S3, respectively.

### NR_027324 was the most consistently upregulated lncRNA throughout timeline and models

**Figure 2:**
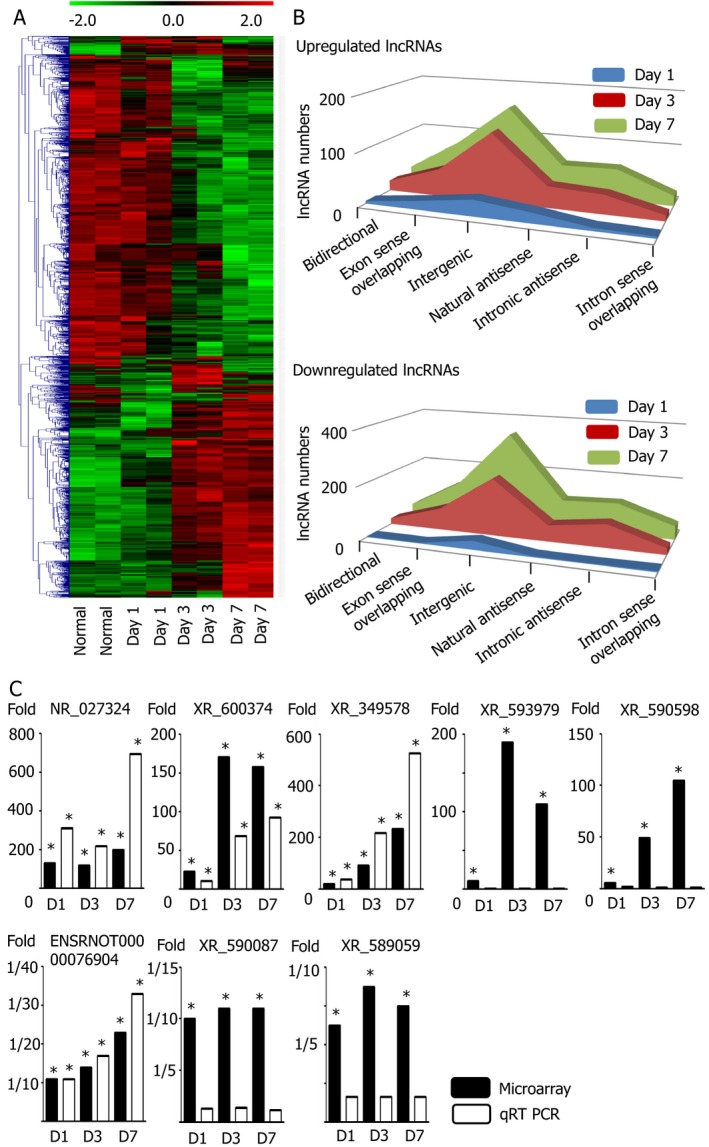
The expression levels of lncRNAs after intracerebral hemorrhage. (A) Heatmap analysis of significantly altered lncRNAs expression patterns from the collagenase‐induced hemorrhagic model showed a distinct temporal pattern of lncRNA. (B) The intergenic lncRNA subfamily is the most up/downregulated group among the six lncRNA subfamilies throughout the three time points. (C) The most elevated lncRNA in the ICH model was NR_027324, which remained elevated until the 7th day after ICH. Among several downregulated lncRNAs, only ENSRNOT00000076904 was consistently found to be decreased by qRT‐PCR. The black bar represents fold changes after collagenase induced ICH on the reference of normal healthy brain derived from microarray analysis; the white bar represents those fold changes derived from qRT‐PCR. Relative fold changes at each time point compared to normal brain from microarray analysis are illustrated in Tables 1 and 2. Relative fold changes (*P* values) from qRT‐PCR are illustrated in Table S4. Each group consists of four individual rat brains; * stands for *P* < 0.05 by Mann–Whitney *U*‐test.

Hierarchical clustering with heatmap by including significantly up/downregulated lncRNAs at each time point revealed characteristic temporal lncRNA expression patterns in the CICH model (Fig. 2A). Among these lncRNAs, the most significantly altered lncRNA subfamily was intergenic, which showed robust increment or decrement at every time point (Fig. 2B). The most upregulated lncRNA 1 day after ICH was NR_027324 and its elevation remained over a hundred‐fold compared to the normal healthy brain until 7 days after ICH (Table 1). The validation by qRT‐PCR showed a similar degree of upregulation after ICH (Fig. 2C, Table S4). The most elevated lncRNA from the BICH model was also NR_027324 (Fig. S1). Gene database analysis revealed that NR_027324, another name for H19, is an intergenic lncRNA which is located in chromosome 11 nearby the Tnnt3 gene which codes troponin T3 fast skeletal type (TNNT3) protein and consists of 2369 base pairs. Other significantly increased lncRNAs from both ICH models were XR_600374, XR_349578, XR_590598, XR_593979, and XR_590598 (Table 1). However, only XR_600374 and XR_349578 showed consistent elevation by qRT‐PCR (Fig. 2C). XR_600374 is an intronic antisense lncRNA located in chromosome 10 with 1469 base pairs, whereas XR_349578 is an exon sense‐overlapping lncRNA located in chromosome 1 with 340 base pairs (Table 1). The expression levels of XR_593979 and XR_590598 were not significantly altered after ICH when examined by qRT‐PCR (Fig. 2C).

**Table 1:** A list of significantly elevated lncRNAs after collagenase‐induced intracerebral hemorrhage.

Sequence name (Gene symbol)	Location (base pairs)	Type	Fold change from microarray (*P*‐value)			
			Day 1	Day 3	Day7	Blood injection model
NR_027324 (H19)	Chr11, 2369	Intergenic	130.2 (0.000090)	120.1 (0.018)	197.8 (0.0012)	82.5 (0.019)
XR_600374 (Igsf7)	Chr10, 1469	intronic antisense	22.9 (0.0012)	170.7 (0.076)	157.8 (0.00040)	27.5 (0.024)
XR_349578 (Lilrb4)	Chr1, 340	exon sense‐overlapping	20.7 (0.0030)	95.5 (0.0031)	232.7 (0.00087)	35.9 (0.017)
XR_590951 (LOC103691483)	Chr2, 687	Intergenic	11.0 (0.011)	53.2 (0.00019)	97.5 (0.0016)	19.1 (0.00032)
XR_593979 (LOC103693120)	Chr8, 377	bidirectional	10.6 (0.0039)	190.1 (0.0024)	110.3 (0.0029)	8.6 (0.023)
ENSRNOT00000076358 (Rn50_X_0663.2)	ChrX, 728	Intergenic	7.2 (0.037)	23.2 (0.011)	27.3 (0.017)	10.2 (0.047)
XR_590598 (LOC103690088)	Chr1, 691	Intergenic	6.3 (0.0063)	49.1 (0.0032)	104.8 (0.0011)	13.9 (0.031)
XR_592669 (LOC102549668)	Chr5, 1239	Intergenic	3.68 (0.024)	58.7 (0.0024)	40.0 (0.0030)	4.51 (0.095)

### ENSRNOT00000076904 was the most downregulated lncRNA after ICH

The most downregulated lncRNA from the CICH model was ENSRNOT00000076904, and its expression was validated by qRT‐PCR (Fig. 2C), but it was not significantly downregulated in the BICH model (Table 2). Other downregulated lncRNAs from the two ICH models were XR_590087 and XR_589059, but their expression levels were not consistently decreased according to qRT‐PCR validation (Fig. 2C). The three aforementioned lncRNAs fall into the intergenic subfamily, but none of them had been previously studied or reported from in vivo experiment.

**Table 2:** A list of significantly decreased lncRNAs after collagenase induced intracerebral hemorrhage.

Sequence name (Gene symbol)	Location (base pairs)	Type	Fold/change from microarray (*P*‐value)			
			Day 1	Day 3	Day 7	Blood injection model
ENSRNOT00000076904 (Irs4)	ChrX, 6299	Intergenic	10.5 (0.023)	13.8 (0.037)	22.8 (0.020)	2.2 (0.15)
XR_590087 (LOC103691048)	Chr1, 3765	intergenic	9.5 (0.0035)	10.8 (0.000082)	10.8 (0.000080)	10.7 (0.000082)
XR_597175 (LOC102553117)	Chr19, 577	Intergenic	8.5 (0.044)	2.1 (0.19)	1.2 (0.49)	3.4 (0.015)
XR_589059 (LOC103690442)	Chr8, 575	intron sense‐overlapping	5.4 (0.012)	6.9 (0.015)	5.5 (0.025)	5.4 (0.025)
XR_589189 (LOC102547709)	Chr9, 972	intergenic	1.1 (0.79)	1.9 (0.23)	5.3 (0.024)	5.6 (0.025)
XR_590402 (LOC103691200)	Chr1, 924	intronic antisense	2.5 (0.23)	2.3 (0.31)	4.1 (0.028)	4.1 (0.028)

### Gene ontology/pathway analysis revealed inflammation as a key biological process after hemorrhagic stroke

**Figure 3:**
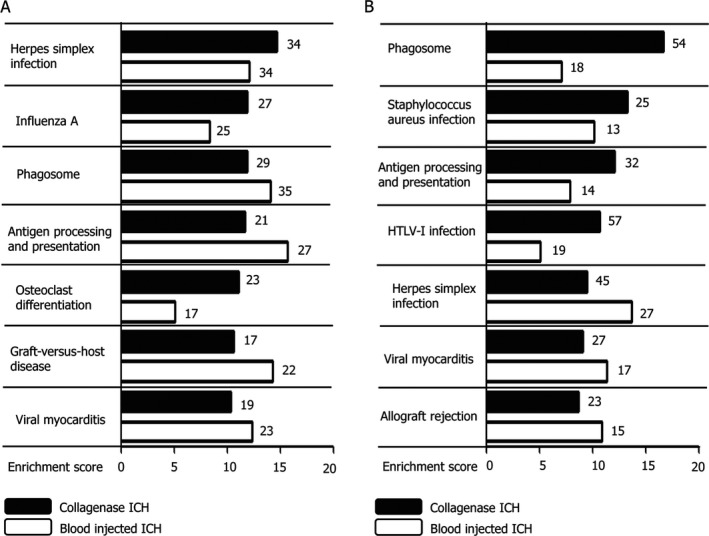
Pathway analysis from two different intracerebral hemorrhage models. (A) Pathway analysis with increased RNAs in the collagenase‐induced ICH model after 1 day showed Herpes simplex infection as the most significantly involved pathway, which includes 34 genes. From the blood injection ICH model, the most significantly involved pathway was antigen processing and presentation which included 27 genes. (B) Three days after ICH, phagosome was the most enriched pathway in the collagenase‐induced model and Herpes simplex infection in the blood‐injected model. The black bars depict enrichment scores from the collagenase‐induced ICH model, and the white bars represent those values from the blood injection ICH model. The right‐sided numbers are gene numbers directly associated with the listed pathway.

The most significantly elevated mRNAs from both ICH models are related with chemokine proteins such as C–X–C motif ligands, secreted phosphoprotein 1, or C–C motif ligands (Table 3). Gene ontology analysis by including significantly upregulated mRNAs after ICH revealed that the biological processes with the highest enrichment score from CICH were immune response, immune system process, and defense response, which were significantly enriched until the 7th day following ICH (Table 4). Similar trends of gene activation patterns were observed from the BICH model, such as immune response and defense mechanism as the primary enriched biological processes (Fig. S2). The cellular components with the highest enrichment scores after CICH were the extracellular region, cell surface, and extracellular region part (Table 4). For molecular function domains, chemokine receptor binding, protein binding, and chemokine activity were major enriched domains after CICH (Table 4). Pathway analysis showed that Herpes simplex infection was the most upregulated pathway in the CICH model, followed by influenza A, phagosome and antigen processing and presentation (Fig. 3A). The main enriched biological pathways from BICH were similar to those from CICH, including antigen processing and presentation with the highest enrichment score, followed by graft‐versus‐host disease, phagosome, and herpes simplex infection (Fig. 3A). The major enriched pathways that remained 3 days after ICH were also immune‐related functions, including phagosome and Herpes simplex infection, with the highest enrichment score from CICH and BICH, respectively (Fig. 3B).

**Table 3:** Top 10 significantly elevated messenger RNAs 1 day after intracerebral hemorrhage.

Collagenase ICH			Blood injection ICH		
Sequence name (Gene symbol)	Fold change	*P* (FDR)	Sequence name (Gene symbol)	Fold change	*P* (FDR)
NM_053647 (Cxcl2)	280.7	0.007 (0.336)	NM_001017496 (Cxcl13)	149.9	0.028 (0.271)
NM_030845 (Cxcl1)	173.7	0.002 (0.297)	NM_012881 (Spp1)	122.8	0.004 (0.248)
NM_012881 (Spp1)	147.6	0.001 (0.272)	ENSRNOT00000075706 (AABR06066155.1)	72.8	0.012 (0.250)
NM_013025 (Ccl3)	136.0	0.009 (0.348)	ENSRNOT00000076919 (Slfn3)	65.0	0.020 (0.261)
NM_031530 (Ccl2)	124.3	0.004 (0.321)	NM_001012029 (Mnda)	60.3	0.029 (0.271)
NM_0011058 (Ccl12)	97.5	0.003 (0.305)	NM_001168285 (RGD1559482)	57.5	0.014 (0.257)
NM_0010076 (Ccl7)	59.7	0.003 (0.321)	NM_053288 (Orm1)	55.8	0.015 (0.257)
ENSRNOT00000075706 (AABR06066155.1)	58.6	0.0002 (0.272)	ENSRNOT00000073646 (Igsf7)	45.6	0.020 (0.261)
ENSRNOT00000056608 (Scimp)	48.7	0.006 (0.336)	NM_001105971 (Slamf9)	45.4	0.017 (0.259)
NM_001168285 (RGD1559482)	45.2	0.0003 (0.272)	ENSRNOT00000075509 (LOC100909671)	44.0	0.017 (0.259)

ICH, intracerebral hemorrhage; FDR, false discovery rate.

**Table 4:** Gene ontology analysis for upregulated genes following collagenase‐induced intracerebral hemorrhage.

	Biological process (ES)	Cellular component (ES)	Molecular function (ES)
Day 1			
1	Immune response (61.4)	Extracellular region (15.9)	Chemokine receptor binding (12.6)
2	Immune system process (59.0)	Cell surface (15.1)	Protein binding (12.2)
3	Defense response (53.9)	Extracellular region part (15.1)	Chemokine activity (10.8)
4	Response to other organism (43.5)	Extracellular space (13.4)	Cytokine activity (10.2)
5	Response to external biotic stimulus (42.9)	External side of plasma membrane (11.0)	CCR chemokine receptor binding (10.1)
Day 3			
1	Immune system process (72.5)	Extracellular region part (27.1)	Protein binding (17.9)
2	Immune response (67.5)	Extracellular region (25.0)	Binding (9.3)
3	Regulation of immune system process (50.9)	Extracellular organelle (22.4)	Receptor binding (9.0)
4	Defense response (50.7)	Extracellular exosome (21.9)	Signaling pattern recognition receptor activity (8.5)
5	Response to stress (46.1)	Extracellular vesicle (21.5)	Pattern recognition receptor activity (8.5)
Day 7			
1	Immune system process (78.1)	Extracellular region part (30.1)	Protein binding (20.1)
2	Immune response (65.4)	Extracellular organelle (28.0)	Protein complex binding (11.2)
3	Regulation of immune system process (51.1)	Extracellular exosome (27.4)	Receptor binding (10.7)
4	Defense response (45.4)	Extracellular vesicle (27.2)	Peptide antigen binding (8.6)
5	Positive regulation of immune system process (44.2)	Extracellular region (26.8)	Chemokine receptor binding (8.5)

ES, enrichment score.

### H19 expression is related with the type I interferon signaling pathway

**Figure 4:**
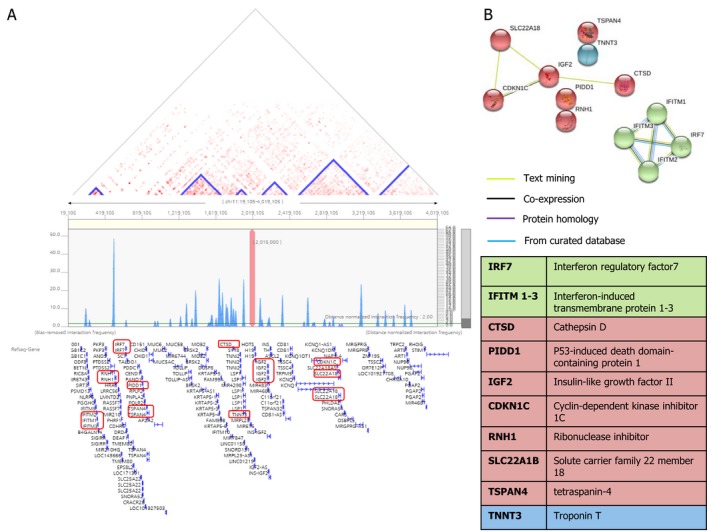
H19 related gene/protein interaction study. (A) Three‐dimensional gene interaction viewer and database (3DIV) analysis revealed that H19 is located in chromosome 11 and related to many protein coding sequences with six topologically associating domains. Those protein sequences which were significantly elevated in the intracerebral hemorrhage model were marked with a red box. (B) STRING analysis showed that the elevated proteins related to H19 after ICH consist of two main groups; one harboring interferon regulatory factor 7 and interferon‐induced transmembrane protein 1–3, which are associated with the type I interferon response pathway, and the other group loosely related to cell survival and proliferation.

3DIV analysis revealed that many protein coding sequences in chromosome 11 are predicted to have a significant association with H19 expression (Fig. 4A). Among those, mRNAs from 12 coding sequences were significantly elevated from the ICH model (marked with red box in Fig. 4A). When those 12 proteins were included in STRING analysis with clustering of k‐means, there existed two main clusters: one cluster including interferon regulatory factor 7 and interferon‐induced transmembrane protein 1‐3 with strong multilevel connections (green group), and the other cluster was related to cell survival/transcriptional signaling with cathepsin D, p53‐induced death domain‐containing protein 1, insulin‐like growth factor 2, cyclin‐dependent kinase inhibitor 1C, ribonuclease inhibitor, solute carrier family 22 member 18 and tetraspanin‐4 (red group, Fig. 4B). TNNT3 was the closest coding sequence to H19, but it did not engage with the two primary clusters.

## Discussion

This study illustrates the lncRNA expression pattern of ICH models and found that intergenic lncRNAs undergo the most vigorous expression alteration during the disease process. By applying both microarray and qRT‐PCR from two different ICH models, we found that H19 is the most upregulated lncRNA after ICH, which remained significant up to 7 days following hemorrhage. The most widely encountered gene expressions after ICH were found to be immune system‐related biological processes, the extracellular region, and chemokine/protein binding, suggesting inflammation as a primary mechanism of neuronal injury after hemorrhagic stroke. Gene and protein interaction analyses predicted H19 elevation is related to the type I interferon signaling pathway after ICH.

The number of significantly elevated and decreased lncRNAs increased as time passed following ICH, suggesting the dynamic nature of the lncRNA expression machinery induced by ICH. Recent studies have shown the therapeutic and biomarker potentials of noncoding RNA expression from various vascular disease conditions including cardiac diseases and ischemic stroke.^12^, ^13^, ^14^, ^15^ LncRNAs are known to regulate multiple biological pathways by epigenetic regulation of gene expression.^12^ Our study showed that intergenic lncRNA undergoes the most dynamic alteration after ICH among the lncRNA subfamilies. This finding does not necessarily confirm its functional role or utility as a therapeutic target against ICH because the majority of lncRNAs belong to an intergenic category, and the mechanism of lncRNA modulating gene expression is known to be diverse.^12^, ^13^ A previous study showed that 359 lncRNAs were upregulated in a 60‐minute transient middle cerebral artery occlusion model and stroke responsive lncRNAs were highly conserved with their protein‐coding genes, but none of them coded proteins.^15^ Another study from a heart failure animal model reported that a cardiac‐specific lncRNA named myosin heavy chain‐associated RNA transcripts protects stress‐induced pathological hypertrophy by repressing the expression of ATP‐dependent chromatin remodeling factors.^16^ Nuclear paraspeckle assembly transcript 1 was the most upregulated lncRNA from both the Huntington's disease animal model and the human postmortem brain, but its transfection into neuro2A cells enhanced cellular survival under oxidative stress, suggesting a defensive role in this pathologic condition.^17^ Regarding hemorrhagic stroke model, lncRNAs expression patterns were investigated during the recovery phase from the rat ICH model, and the authors reported that mitochondrial matrix, reduced G‐protein coupled receptor activity, and impaired olfactory transduction could be therapeutic targets to facilitate functional recovery based on gene ontology analysis.^18^ Another group reported 144 downregulated and 64 upregulated lncRNAs from subarachnoid hemorrhage model.^19^ Whether significantly elevated lncRNA from this study could be a potential target of therapeutic intervention requires future studies.

The most upregulated lncRNA from both CICH and BICH was NR_027324, which was also known as H19, and had been extensively studied from neoplastic disorders.^20^ H19 is one of the most highly expressed lncRNAs during embryonal development and is downregulated after birth, and broadly conserved throughout species.^20^ Hypoxia can elicit the expression of H19, and a recent study showed an elevation of H19 expression in cerebral infarction.^21^ This study showed that H19 expression was both elevated in peripheral blood of acute infarction patients and correlated with their neurological severity, and H19 promotes neuroinflammation by driving histone deacetylase 1‐dependent microglial polarization from the animal model.^21^ Another recent study showed significant upregulation of H19 during the latent period of temporal lobe epilepsy animal model, and H19 might function as competing endogenous RNA to sponge let7b in the regulation of cellular apoptosis.^22^ Our group previously reported that upregulated let7c in the ICH model facilitated inflammation and apoptotic cell death, and its inhibition by an antagonistic sequence enhanced neurological recovery.^7^ Whether the elevation of H19 after hemorrhage has a defensive role against neuronal damage by let7, or it is an independent factor of neuronal injury warrants further study.

Our study suggests that the immune response could be a promising therapeutic target after ICH based on gene ontology and pathway analyses. Gene ontology analysis revealed consistent upregulation of genes related to immune response and immune system process in both ICH models. Pathway analysis showed that genes related to herpes simplex infection, phagosome, and antigen processing and presentation could be a potential target. Previous studies have shown that rapid upregulation of inflammatory proteins after ICH from animal model studies and its modulation exhibited a neuroprotective effect.^23^, ^24^ Recently a small‐sized clinical study reported that celecoxib treatment could reduce peri‐hematoma brain edema among hemorrhagic stroke patients without significant adverse effects.^25^ Another study examined the efficacy of fingolimod, an anti‐inflammatory therapeutic agent for the prevention of multiple sclerosis progression, could also reduce neurological injury after ICH by reducing brain edema.^26^ Our study results support the application of an anti‐inflammatory strategy in ICH treatment, with a prolonged therapeutic window up to 7 days.

Protein interaction study from STRING analysis revealed that the proteins transcribed by the dysregulated mRNAs related to H19 after ICH were involved in the type I interferon signaling pathway. Interferons play essential roles in establishing and modulating host defense against microbial infection, but dysregulation of interferon production or its function could mediate a pathologic immune response or host tissue damage.^27^ Interferon regulatory factor 7 is ubiquitously expressed by most cell types and organs, and positively regulates the Toll‐like receptor‐dependent pro‐inflammatory response.^28^ Interferon‐induced transmembrane proteins are another key host molecules of the type I interferon response against viruses, but they also participate in the control of cell proliferation, promotion of homotypic cell adhesion, bone matrix formation, and mediating germ cell development.^29^ A previous study demonstrated that interferon regulatory factor 7 is an important molecule related to the neuroprotective effect of ischemic preconditioning.^30^ Recently, a clinical trial demonstrated that a monoclonal antibody against the interleukin‐1*β*/6 signaling pathway successfully reduced recurrent atherosclerotic vascular events.^31^ Whether a similar strategy focusing on inflammatory cytokines can be applied to hemorrhagic stroke patients needs to be studied because hemorrhagic stroke is also associated with excessive localized and systemic immune reactions.^32^

Several limitations exist in the study. Although our study showed that H19 as the most elevated lncRNA from two ICH models, the direct mechanistic link between H19 elevation and inflammatory injury is not sufficient. Future study with in vitro genetic modification or in situ hybridization is necessary to disclose how H19 participates in neuronal injury in ICH. There are other classes of noncoding RNAs such as microRNAs and circular RNAs which can influence protein coding, and lncRNAs can also regulate mRNA expression by competitively binding to microRNAs.^33^ However, we did not take into account the interaction of lncRNAs with other noncoding RNAs. A recent study disclosed that male and female stroke patients have differential expression pattern of lncRNAs located near stroke‐risk genes.^34^ The neuronal damage mechanism based on sexual dimorphism could not be evaluated in this study because only male rats had been used. We chose normal controls which had not been through any surgical procedure, but the sham rats treated with stereotactic needle insertion might be better controls because diverse neuronal damage can elicit distinct genomic responses.^35^ GAPDH was used as a house‐keeping gene for qRT‐PCR, although its expression could be variable according to different stroke models.^36^

This study disclosed the dynamic expression patterns of lncRNA from two different ICH animal models and derived a significantly upregulated lncRNA which might be a therapeutic target after ICH. Whether treatment strategies focusing on inflammation modulation can alleviate hemorrhage‐induced neuronal injury warrants future investigation.

## Author Contributions

Kim JM involved in drafting a significant portion of the manuscript and figures; Moon J, Yu JS, and Park DK involved in acquisition and analysis of data; Lee ST, Jung KH, and Chu K involved in conception and design of the study.

## Conflict of Interest

None.

## Supporting information


**Figure S1.** Quantitative RT‐PCR validation of lncRNA expression from the blood injected ICH model.

**Figure S2.** Gene ontology analysis results from the blood injected ICH model.

**Table S1.** Primer sequences for qRT‐PCR validations.

**Table S2.** The expression levels of long noncoding RNAs in rat hemorrhagic stroke models.

**Table S3.** The expression levels of messenger RNAs in rat hemorrhagic stroke models.

**Table S4.** Relative fold changes and *P* values from quantitative RT‐PCR.

## References

[1] An SJ , Kim TJ , Yoon BW . Epidemiology, risk factors, and clinical features of intracerebral hemorrhage: an update. J Stroke 2017;19:3–10. 28178408 10.5853/jos.2016.00864 PMC5307940

[2] Kim JY , Bae HJ . Spontaneous intracerebral hemorrhage: management. J Stroke 2017;19:28–39. 28178413 10.5853/jos.2016.01935 PMC5307946

[3] Ma L , Bajic VB , Zhang Z . On the classification of long non‐coding RNAs. RNA Biol 2013;10:925–933. 23696037 10.4161/rna.24604 PMC4111732

[4] Lee DY , Moon J , Lee ST , et al. Distinct expression of long non‐coding RNAs in an Alzheimer's disease model. J Alzheimers Dis 2015;45:837–849. 25624420 10.3233/JAD-142919

[5] Lee DY , Moon J , Lee ST , et al. Dysregulation of long non‐coding RNAs in mouse models of localization‐related epilepsy. Biochem Biophys Res Commun 2015;462:433–440. 25976677 10.1016/j.bbrc.2015.04.149

[6] Zhang X , Tang X , Liu K , et al. Long noncoding RNA Malat1 regulates cerebrovascular pathologies in ischemic stroke. J Neurosci 2017;37:1797–1806. 28093478 10.1523/JNEUROSCI.3389-16.2017 PMC5320610

[7] Kim JM , Lee ST , Chu K , et al. Inhibition of let7c microRNA is neuroprotective in a rat intracerebral hemorrhage model. PLoS ONE 2014;9:e97946. 24959881 10.1371/journal.pone.0097946 PMC4068982

[8] Matsushita K , Meng W , Wang X , et al. Evidence for apoptosis after intracerebral hemorrhage in rat striatum. J Cereb Blood Flow Metab 2000;20:396–404. 10698078 10.1097/00004647-200002000-00022

[9] Engreitz JM , Haines JE , Perez EM , et al. Local regulation of gene expression by lncRNA promoters, transcription and splicing. Nature 2016;539:452–455. 27783602 10.1038/nature20149 PMC6853796

[10] Yang D , Jang I , Choi J , et al. 3DIV: a 3D‐genome interaction viewer and database. Nucleic Acids Res 2018;46:D52–D57. 29106613 10.1093/nar/gkx1017 PMC5753379

[11] Szklarczyk D , Morris JH , Cook H , et al. The STRING database in 2017: quality‐controlled protein‐protein association networks, made broadly accessible. Nucleic Acids Res 2017;45:D362–D368. 27924014 10.1093/nar/gkw937 PMC5210637

[12] Engreitz JM , Ollikainen N , Guttman M . Long non‐coding RNAs: spatial amplifiers that control nuclear structure and gene expression. Nat Rev Mol Cell Biol 2016;17:756–770. 27780979 10.1038/nrm.2016.126

[13] Jang Y , Moon J , Lee ST , et al. Dysregulated long non‐coding RNAs in the temporal lobe epilepsy mouse model. Seizure 2018;58:110–119. 29702408 10.1016/j.seizure.2018.04.010

[14] Devaux Y , Zangrando J , Schroen B , et al. Long noncoding RNAs in cardiac development and ageing. Nat Rev Cardiol 2015;12:415–425. 25855606 10.1038/nrcardio.2015.55

[15] Dharap A , Nakka VP , Vemuganti R . Effect of focal ischemia on long noncoding RNAs. Stroke 2012;43:2800–2802. 22949471 10.1161/STROKEAHA.112.669465 PMC3458128

[16] Han P , Li W , Lin CH , et al. A long noncoding RNA protects the heart from pathological hypertrophy. Nature 2014;514:102–106. 25119045 10.1038/nature13596 PMC4184960

[17] Sunwoo JS , Lee ST , Im W , et al. Altered expression of the long noncoding RNA NEAT1 in Huntington's disease. Mol Neurobiol 2017;54:1577–1586. 27221610 10.1007/s12035-016-9928-9

[18] Hanjin C , Tao L , Pengfei L , et al. Altered long noncoding RNA and messenger RNA expression in experimental intracerebral hemorrhage ‐ a preliminary study. Cell Physiol Biochem 2018;45:1284–1301. 29448258 10.1159/000487464

[19] Zheng B , Liu H , Wang R , et al. Expression signatures of long non‐coding RNAs in early brain injury following experimental subarachnoid hemorrhage. Mol Med Rep 2015;12:967–973. 25777551 10.3892/mmr.2015.3474 PMC4438960

[20] Kiang KM , Zhang XQ , Leung GK . Long non‐coding RNAs: the key players in glioma pathogenesis. Cancers (Basel) 2015;7:1406–1424. 26230711 10.3390/cancers7030843 PMC4586776

[21] Wang J , Zhao H , Fan Z , et al. Long noncoding RNA H19 promotes neuroinflammation in ischemic stroke by driving histone deacetylase 1‐dependent M1 microglial polarization. Stroke 2017;48:2211–2221. 28630232 10.1161/STROKEAHA.117.017387

[22] Han CL , Ge M , Liu YP , et al. LncRNA H19 contributes to hippocampal glial cell activation via JAK/STAT signaling in a rat model of temporal lobe epilepsy. J Neuroinflammation 2018;15:103. 29636074 10.1186/s12974-018-1139-z PMC5894243

[23] Mracsko E , Veltkamp R . Neuroinflammation after intracerebral hemorrhage. Front Cell Neurosci 2014;8:388. 25477782 10.3389/fncel.2014.00388 PMC4238323

[24] Kim JM , Lee ST , Chu K , et al. Systemic transplantation of human adipose stem cells attenuated cerebral inflammation and degeneration in a hemorrhagic stroke model. Brain Res 2007;1183:43–50. 17920570 10.1016/j.brainres.2007.09.005

[25] Lee SH , Park HK , Ryu WS , et al. Effects of celecoxib on hematoma and edema volumes in primary intracerebral hemorrhage: a multicenter randomized controlled trial. Eur J Neurol 2013;20:1161–1169. 23551657 10.1111/ene.12140

[26] Fu Y , Hao J , Zhang N , et al. Fingolimod for the treatment of intracerebral hemorrhage: a 2‐arm proof‐of‐concept study. JAMA Neurol 2014;71:1092–1101. 25003359 10.1001/jamaneurol.2014.1065

[27] Chen K , Liu J , Cao X . Regulation of type I interferon signaling in immunity and inflammation: a comprehensive review. J Autoimmun 2017;83:1–11. 28330758 10.1016/j.jaut.2017.03.008

[28] Zhang XJ , Jiang DS , Li H . The interferon regulatory factors as novel potential targets in the treatment of cardiovascular diseases. Br J Pharmacol 2015;172:5457–5476. 25131895 10.1111/bph.12881 PMC4667854

[29] Hickford D , Frankenberg S , Shaw G , Renfree MB . Evolution of vertebrate interferon inducible transmembrane proteins. BMC Genom 2012;13:155. 10.1186/1471-2164-13-155 PMC3424830 22537233

[30] Stevens SL , Leung PY , Vartanian KB , et al. Multiple preconditioning paradigms converge on interferon regulatory factor‐dependent signaling to promote tolerance to ischemic brain injury. J Neurosci 2011;31:8456–8463. 21653850 10.1523/JNEUROSCI.0821-11.2011 PMC3130521

[31] Ridker PM , Everett BM , Thuren T , et al. Antiinflammatory therapy with canakinumab for atherosclerotic disease. N Engl J Med 2017;377:1119–1131. 28845751 10.1056/NEJMoa1707914

[32] Ye L , Gao L , Cheng H . Inflammatory profiles of the interleukin family and network in cerebral hemorrhage. Cell Mol Neurobiol 2018;38:1321–1333. 30027390 10.1007/s10571-018-0601-x PMC11481843

[33] Salmena L , Poliseno L , Tay Y , et al. A ceRNA hypothesis: the Rosetta stone of a hidden RNA language? Cell 2011;146:353–358. 21802130 10.1016/j.cell.2011.07.014 PMC3235919

[34] Dykstra‐Aiello C , Jickling GC , Ander BP , et al. Altered expression of long noncoding RNAs in blood after ischemic stroke and proximity to putative stroke risk loci. Stroke 2016;47:2896–2903. 27834745 10.1161/STROKEAHA.116.013869 PMC5127755

[35] Tang Y , Lu A , Aronow BJ , Sharp FR . Blood genomic responses differ after stroke, seizures, hypoglycemia, and hypoxia: blood genomic fingerprints of disease. Ann Neurol 2001;50:699–707. 11761467 10.1002/ana.10042

[36] Kang Y , Wu Z , Cai, D , Lu B . Evaluation of reference genes for gene expression studies in mouse and N2a cell ischemic stroke models using quantitative real‐time PCR. BMC Neurosci 2018;19:3. 29390963 10.1186/s12868-018-0403-6 PMC5795833

