# Supplementary figures and images for: Altered long noncoding RNA profile after intracerebral hemorrhage
Source: Ann Clin Transl Neurol. 2019 Sep 26;6(10):2014–25. doi: 10.1002/acn3.50894 (PMC6801204; doi:10.1002/acn3.50894)

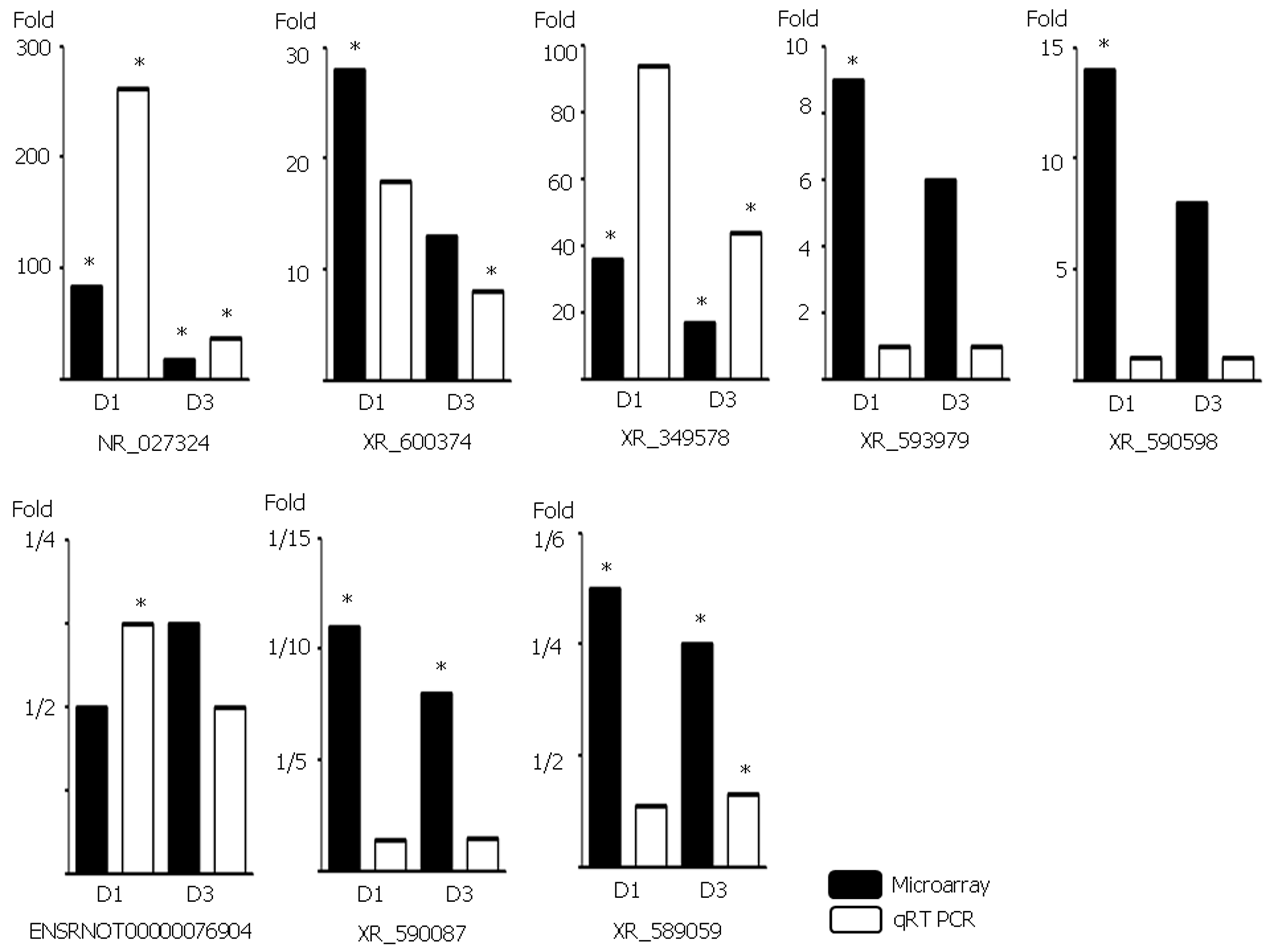

Supplement: Supplementary file 1 — Figure S1. Quantitative RT‐PCR validation of lncRNA expression from the blood injected ICH model. [file ACN3-6-2014-s001.tif]

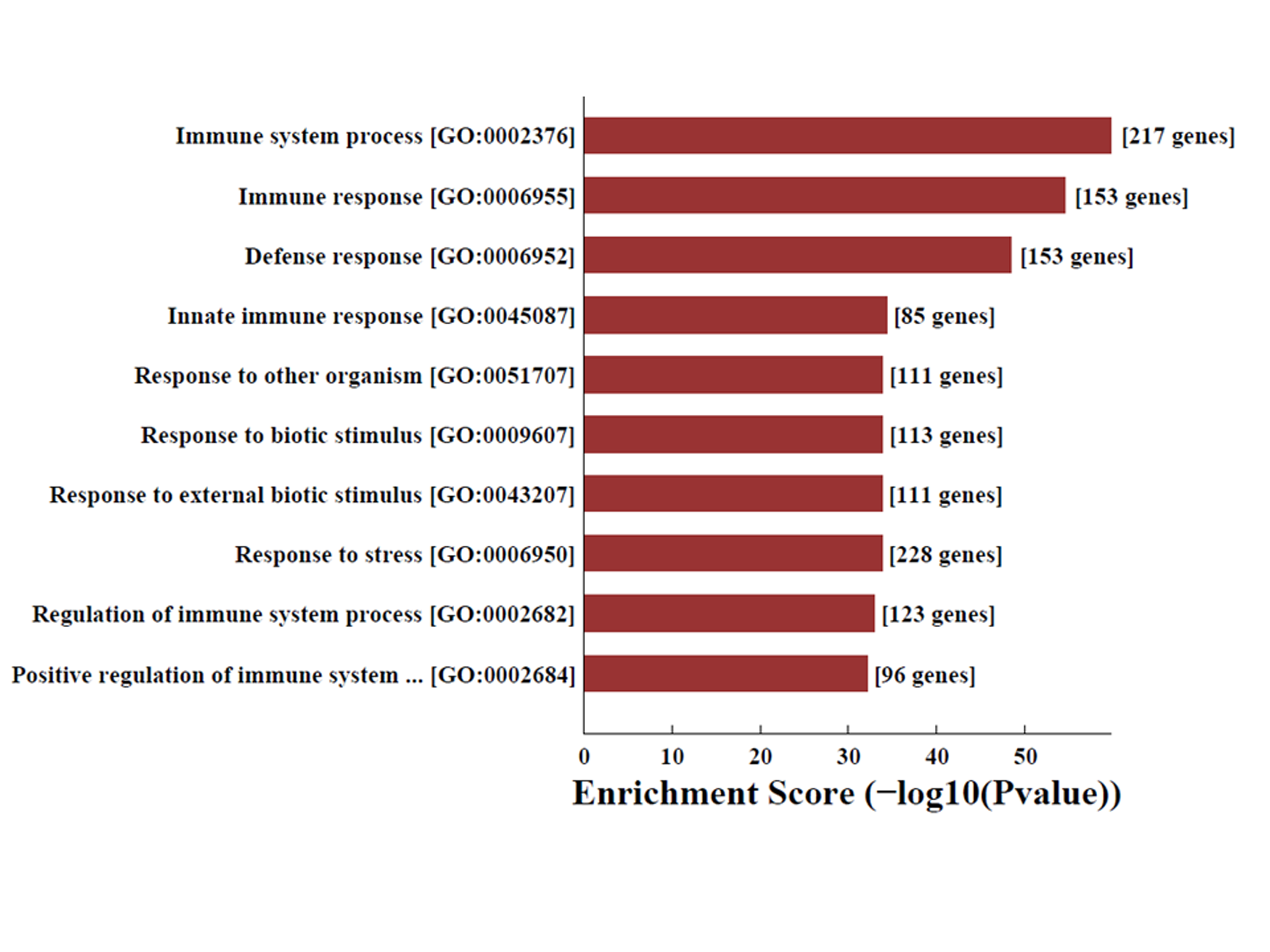

Supplement: Supplementary file 2 — Figure S2. Gene ontology analysis results from the blood injected ICH model. [file ACN3-6-2014-s002.tif]
