# Supplementary material for: Altered long noncoding RNA profile after intracerebral hemorrhage
Source: Ann Clin Transl Neurol. 2019 Sep 26;6(10):2014–25. doi: 10.1002/acn3.50894 (PMC6801204; doi:10.1002/acn3.50894)
Supplement: Supplementary file 3 — Table S1. Primer sequences for qRT‐PCR validations. [file ACN3-6-2014-s003.docx]

1. Supplementary table 1. The primers’ sequence information

| Gene Name | Sequence (5’ to 3’) | Tm(°C) | Length in base pairs |
| --- | --- | --- | --- |
| GAPDH | Forward:5’ GCTCTCTGCTCCTCCCTGTTCTA 3'  Reverse:5’ TGGTAACCAGGCGTCCGATA 3’ | 60 | 124 |
| NR_027324 | Forward:5’ TGAATCCGGGGACTTCTTTAAG 3'  Reverse:5’ GGTGCTGTGTGGGTCTGCT 3’ | 60 | 154 |
| XR_600374 | Forward:5’ CCATGTGAAGCAGAGTGACAGA 3'  Reverse:5’ CTGTTGTAATGCTCCAAAGGCT 3’ | 60 | 123 |
| XR_349578 | Forward:5’ CTGTCCATAGATTAAAGCCTCC 3'  Reverse:5’ AGTGCTGATCCAAGACCCTC 3’ | 60 | 190 |
| XR_593979 | Forward:5’ AACTGGAAGTGTCAATGTGCC 3'  Reverse:5’ GGAGATAGGGAAAGGTCTGAGTA 3’ | 60 | 111 |
| XR_590598 | Forward:5’ CATTAGTAATCAAGGCAGTTCAT 3'  Reverse:5’ CAGTGCTGAGAGAGTGAGGTGT 3’ | 60 | 209 |
| ENSRNOT00000076904 | Forward:5’ TGAGGACAACTATGACGATGAG 3'  Reverse:5’ TGCTTGTTGACTGGTAGAAATC 3’ | 60 | 245 |
| XR_590087 | Forward:5’ TTCTTTACCGCCTCTTGTTCC 3'  Reverse:5’ TGTGCTACTGTGTACCGTCTGG 3’ | 60 | 65 |
| XR_589059 | Forward:5’ TGTCCATCGTGCTATCCCT 3'  Reverse:5’ GCAGTAGGAGACAAAACGAAAG 3’ | 60 | 143 |
