# Supplementary material for: Altered long noncoding RNA profile after intracerebral hemorrhage
Source: Ann Clin Transl Neurol. 2019 Sep 26;6(10):2014–25. doi: 10.1002/acn3.50894 (PMC6801204; doi:10.1002/acn3.50894)
Supplement: Supplementary file 6 — Table S4. Relative fold changes and P values from quantitative RT‐PCR. [file ACN3-6-2014-s006.docx]

Supplementary table 4. Relative fold changes and p values of quantitative RT-PCR

|  | Collagenase ICH | | | Blood injection ICH | |
| --- | --- | --- | --- | --- | --- |
|  | Day 1  Fold change  (p-value) | Day 3  Fold change  (p-value) | Day 7  Fold change  (p-value) | Day 1  Fold change  (p-value) | Day 3  Fold change  (p-value) |
| NR_027324 | 310.0 (0.029) | 219.6 (0.039) | 659.3 (0.012) | 262.5 (0.034) | 37.3 (0.0053) |
| XR_600374 | 11.2 (0.000071) | 69.1 (0.0080) | 93.2 (0.014) | 18.5 (0.052) | 7.7 (0.004) |
| XR_349578 | 36.7 (0.010) | 218.0 (0.084) | 528.4 (0.0024) | 93.6 (0.075) | 44.1 (0.024) |
| XR_593979 | 1.1 (0.73) | 1.2 (0.20) | 0.92 (0.79) | 0.83 (0.50) | 0.59 (0.068) |
| XR_590598 | 1.6 (0.079) | 1.2 (0.39) | 0.68 (0.54) | 1.4 (0.64) | 0.30 (0.035) |
| ENSRN0T00000076904 | 0.09 (0.013) | 0.06 (0.013) | 0.03 (0.011) | 0.35 (0.027) | 0.48 (0.082) |
| XR_590087 | 0.76 (0.0046) | 0.72 (0.090) | 0.89 (0.0048) | 0.69 (0.24) | 0.65 (0.054) |
| XR_589059 | 0.77 (0.052) | 0.76 (0.066) | 0.78 (0.078) | 0.91 (0.60) | 0.74 (0.034) |

ICH stands for intracerebral hemorrhage.
